# Bacteriophage-Based Biosensing of *Pseudomonas aeruginosa*: An Integrated Approach for the Putative Real-Time Detection of Multi-Drug-Resistant Strains

**DOI:** 10.3390/bios11040124

**Published:** 2021-04-15

**Authors:** Liliam K. Harada, Waldemar Bonventi Júnior, Erica C. Silva, Thais J. Oliveira, Fernanda C. Moreli, José M. Oliveira Júnior, Matthieu Tubino, Marta M. D. C. Vila, Victor M. Balcão

**Affiliations:** 1 PhageLab—Laboratory of Biofilms and Bacteriophages, University of Sorocaba, Sorocaba, SP 18023-000, Brazil; liliam.harada@edu.uniso.br (L.K.H.); erica.silva@edu.uniso.br (E.C.S.); thais.oliveiras@edu.uniso.br (T.J.O.); fernanda.moreli@edu.uniso.br (F.C.M.); jose.oliveira@prof.uniso.br (J.M.O.J.); marta.vila@prof.uniso.br (M.M.D.C.V.); 2 Faculty of Technology of Sorocaba—FATEC SO, Sorocaba, SP 18013-280, Brazil; waldemar.junior@fatec.sp.gov.br; 3 Institute of Chemistry, University of Campinas, Campinas, SP 13083-970, Brazil; tubino@unicamp.br; 4 Department of Biology and CESAM, University of Aveiro, Campus Universitário de Santiago, P-3810-193 Aveiro, Portugal

**Keywords:** bacteriophage particles, *Pseudomonas aeruginosa*, immobilization and structural/functional stabilization, bacterial biosensing, bio-reactive polymeric matrix, chromogenic/bioluminescent bio-hydrogel

## Abstract

During the last decennium, it has become widely accepted that ubiquitous bacterial viruses, or bacteriophages, exert enormous influences on our planet’s biosphere, killing between 4–50% of the daily produced bacteria and constituting the largest genetic diversity pool on our planet. Currently, bacterial infections linked to healthcare services are widespread, which, when associated with the increasing surge of antibiotic-resistant microorganisms, play a major role in patient morbidity and mortality. In this scenario, *Pseudomonas aeruginosa* alone is responsible for ca. 13–15% of all hospital-acquired infections. The pathogen *P. aeruginosa* is an opportunistic one, being endowed with metabolic versatility and high (both intrinsic and acquired) resistance to antibiotics. Bacteriophages (or phages) have been recognized as a tool with high potential for the detection of bacterial infections since these metabolically inert entities specifically attach to, and lyse, bacterial host cells, thus, allowing confirmation of the presence of viable cells. In the research effort described herein, three different phages with broad lytic spectrum capable of infecting *P. aeruginosa* were isolated from environmental sources. The isolated phages were elected on the basis of their ability to form clear and distinctive plaques, which is a hallmark characteristic of virulent phages. Next, their structural and functional stabilization was achieved via entrapment within the matrix of porous alginate, biopolymeric, and bio-reactive, chromogenic hydrogels aiming at their use as sensitive matrices producing both color changes and/or light emissions evolving from a reaction with (released) cytoplasmic moieties, as a bio-detection kit for *P. aeruginosa* cells. Full physicochemical and biological characterization of the isolated bacteriophages was the subject of a previous research paper.

## 1. Introduction

Among the microorganisms that cause hospital infections one can highlight the multidrug-resistant *Pseudomonas aeruginosa*, which represents a major threat to patients in health care services [1,2,3,4]. The surge of multiple bacterial resistance to conventional antibiotics [5,6] has been gaining increasing worldwide awareness, leading to a renewed interest in phage particles by the scientific community, with these entities (devoid of any metabolic machinery) being re-discovered as having high-potential in biopharmaceutical applications, such as antimicrobial or bacterial biosensing [1,2,7,8,9,10,11,12]. Although highly innovative, these proposed concepts are not new, existing since about 3.5 billion years and having been initiated when bacteria and phages established a predator-prey balance. Phages can be found wherever their bacterial hosts exist. The total population of phages is now estimated to be 10^8^ species and 10^31^ particles in the biosphere, which makes them the most plentiful entities on Earth [13,14,15,16]. Phage population is huge and full of novel genes whose functions are essentially unknown [17], and, for this reason, they are considered the “dark matter” of the biological universe [18]. *P. aeruginosa* has evolved over time, becoming progressively more resistant to the available antibiotics, and is now considered one of the six most dangerous pathogens responsible for hospital-acquired infections [19,20,21].

Speed, sensitivity, and specificity are the major requirements needed for methodologies aiming at bacterial pathogen detection. In addition, such methodologies should be robust, cheap, and easy to operate, hence, being feasible for diverse types of samples and conditions [22,23,24]. Requirements are especially important if application of such pathogen detection methodologies are aimed at developing countries, where economic resources are scarce and high mortality rates arise from bacterial infections [25]. Pulmonary infections are usually diagnosed following microscopy observation of patient sputum with confirmation via sputum culture procedures that are neither sensitive or specific. The automated BACTEC^®^ system (Becton, Dickinson, and Company) and FastPlaqueTB™ (BIOTEC Laboratories Ltd., Martlesham, UK) are much more efficient, but the major drawbacks associated with this equipment are, on one hand, their high cost and, on the other hand, the need for laboratory infrastructures, complicating their implementation in most developing countries. In addition, positive cultures take a relatively long timeframe (viz. 24 h) to be detected [26,27]. The TaqMan^®^ (Applied Biosystems, Waltham, MA, USA) detection kit provides a reliable and fast procedure for detecting *P. aeruginosa* cells, but it also requires both expensive equipment and specific reactants. The conventional detection methodologies use selective culture media together with biochemical and serological characterization [25,28,29], offering a high sensitivity, but the need for lengthy sample enrichment and development of bacterial colonies are its major drawbacks.

Therefore, lytic phage particles have been attracting more attention as recognition elements of pathogenic bacterial cells, by taking advantage of the high specificity of phage particles to their bacterial host cells [13,22,25,28,29,30,31,32,33]. The bacterial cell-specific phage-recognition mechanism has evolved naturally, with phage particles specifically recognizing surface receptors on their bacterial host cells and strongly binding to them, hence, being formidable tools for detecting bacterial pathogens [28]. The unique ability of phage particles to specifically infect and kill their bacterial host cells [1,25,30,33,34,35,36,37,38,39,40,41,42], discriminating between viable and non-viable cells, can be exploited for developing assays that detect the release of bacterial cytoplasmic moieties [24,43,44]. Bio-detection of bacterial cells through phage-induced lysis may be attained via colorimetric methodologies using released ATP [45]. Regarding immobilization/stabilization of phage particles, and, despite their resilience to abiotic factors, one must adopt innocuous immobilization procedures such as simple physical immobilization within a biopolymeric hydrogel matrix [7,8,46,47,48]. Although not explored as extensively as other immobilization procedures, immobilization within a biopolymeric hydrogel matrix would allow (at least theoretically) the use of mild polymerization conditions, with concomitant structural and functional stabilization of the phage particles and maintenance of their lytic viability. Research papers reporting immobilization of phage particles in biopolymeric matrices aiming at developing bacterial detection systems are very scarce [7,8,49]. The characteristics of a gelled nano-porous matrix with entrapped phage particles display a high potential for bacterial biosensing applications since, in addition to protecting the phage particles, the semi-solid nano-porous matrix should facilitate its direct contact with the biological fluid sample under study and the contact of any bacterial cells present therein with the immobilized phage particles via bacterial cell diffusion into and within the matrix core [8].

In the present research effort, three different lytic phages for *P. aeruginosa* were immobilized via entrapment within porous biopolymeric and bio-reactive alginate matrices with concomitant full structural and functional stabilization, aiming at their use as sensitive bio-reactive hydrogels producing both color changes and/or light emission evolving from reaction with (released) cytoplasmic moieties, as a bio-detection kit for *P. aeruginosa* cells. Two different approaches were followed, with one involving development of color in a chromogenic bio-hydrogel and the other involving fluorescent light emission, as a result of the presence of viable *P. aeruginosa* cells in a contacting fluid.

## 2. Materials and Methods

### 2.1. Materials

#### 2.1.1. Biological Materials

##### Bacteria

The collection strain of P. aeruginosa (ref. DSM 19880) used as a phage isolation host was acquired from DSMZ (Deutsche Sammlung von Mikroorganismen und Zellkulturen GmmH, Braunschweig, Germany). The bacterial strain of Staphylococcus aureus CCCD-S009, used as control in the bio-detection assays using the chromogenic hydrogel, was purchased from CEFAR (São Paulo, SP, Brazil).

##### Phages

Lytic phages for P. aeruginosa were isolated from samples of sewage water from Hospital UNIMED “Miguel Soeiro” and from SAAE/Sorocaba in Sorocaba/SP, Brazil, collected on 5 September, 2017. One of the phages used (phage JG004, ref. DSM 19871) was acquired from DSMZ (Deutsche Sammlung von Mikroorganismen und Zellkulturen GmmH, Braunschweig, Germany). Phage JG004 was propagated in the same P. aeruginosa host (ref. DSM 19880), maintained at −86 °C following inoculation of a 1.5 mL-cryotube containing Tryptic Soy Broth (TSB) and 30% (*v*/*v*) glycerol. Extensive characterization of the isolated phage particles (ph0031, ph0034, and ph0041), from a physicochemical, biological, and genomic points of view, was the subject of a previous publication by Harada et al. (currently submitted for publication).

#### 2.1.2. Chemicals

The culture media Tryptic Soy Broth (TSB) (ref. SIGMA 22092-500G) and Tryptic Soy Agar (TSA) (ref. SIGMA 22091-500G) were purchased from Sigma-Aldrich (St. Louis, MO, USA) and solid agar was purchased from Gibco Diagnostics (Madison, WI, USA). Nipagin^®^ (methyl-parahydroxibenzoate) (ref. SIGMA 47889), glucono-δ-lactone (GDL) (ref. SIGMA G2164-100G), alginic acid sodium salt from brown algae (ref. SIGMA A2033-250G), D-Luciferin (ref. SIGMA L9504-50MG), luciferase from *Photinus pyralis* (ref. SIGMA SRE0045-10MG), ADP (adenosine 5′-diphosphate sodium salt) (ref. SIGMA A2754-1G), magnesium chloride (ref. SIGMA M8266-100G), casein from bovine milk (ref. SIGMA C6554-500G), gelatin (ref. SIGMA 48723-500G-F), and sodium 1,2-naphthoquinone-4-sulfonate (ref. SIGMA 70382-10G), were all purchased from Sigma-Aldrich (St. Louis, MO, USA). Calcium carbonate (CaCO_3_) (ref. ANIDROL PAP.A-1506) was purchased from ANIDROL (Diadema/SP, Brazil). The sterilizing filtration system used (Stericup™-GP, polyethersulphate (PES) membrane with a 0.22-µm pore diameter) was purchased from Merck-Millipore (Darmstadt, Germany). Tap water was ultra-purified to a final resistivity of ca. 18.18 MΩ·cm and conductivity of 0.05 µS·cm^−1^, in a Master System All (model MS2000, Gehaka, São Paulo, SP, Brazil).

### 2.2. Experimental Procedures

#### 2.2.1. Bacterial Strain and Growth Conditions

The bacterial strain of P. aeruginosa (DSM 19880), used in this study as phage host, was acquired from DSMZ (Braunschweig, Germany). Fresh cultures of bacteria were maintained in TSA at 4 °C and, before each assay, one isolated colony was transferred to 25 mL of TSB (Tryptic Soy Broth) and grown overnight at 37 °C. An aliquot of this culture (100 µL) was then poured in 10 mL of fresh TSB and grown at 37 °C to reach an optical density (OD_600 nm_) of 1.0, corresponding to ca. 10^9^ cells/mL.

#### 2.2.2. Preparation of *P. aeruginosa* Bacterial Lawns

One hundred μL of overnight grown *P. aeruginosa* DSM19880 bacterial suspension were mixed with 5 mL of molten top agar–TSB (MTA-TSB) (maintained at ca. 47 °C) in a 15 mL test tube and tapped gently. The contents of this tube were then poured onto an agar plate with solid TSA, which was gently swirled, and the plates were allowed to dry for 1–2 min.

#### 2.2.3. Gelification/Polymerization Conditions for Producing Sodium Alginate-Based Matrices

For preparing suitable hydrogel matrices aiming at structurally and functionally stabilizing the phage particles, calcium alginate was used on the basis of previous research works [7,8], using the internal gelification method [7,50,51]. As a mold for the several hydrogels, six-well cell culture plates were used, with 2.00 g of polymeric solution being poured into each well. Calcium carbonate was used as the source of calcium ions. The process (Table 1) was initiated by preparing calcium carbonate and sodium alginate dispersions in ca. 80% (*w*/*w*) of the total mass of ultrapure water containing casein, gelatin, sodium-1,2-naphthoquinone-4-sulfonate, phage cocktail, and methylparaben (biodetection system I) or, in the absence of light, luciferin, luciferase, ADP, Mg^2+^, phage cocktail and the antifungal methylparaben (biodetection system II). In formulations 1–3 (Table 1), the phage cocktail encompassed 15 µL of each phage stock suspension (virion amounts: phage ph0031, 6.60 × 10^11^ virions, phage ph0034, 1.80 × 10^10^ virions, phage ph0041, 2.70 × 10^10^ virions), whereas, in formulations 4–5 (Table 1), the phage cocktail encompassed 50 µL of each phage stock suspension (virion amounts: phage ph0031, 2.20 × 10^12^ virions, phage ph0034, 6.00 × 10^10^ virions, phage ph0041, 9.00 × 10^10^ virions). Hence, samples of the phage stock suspensions were mixed and added to the polymeric formulations in a concentration of 0.45% (*w*/*w*) (formulations 1–3, corresponding to approximately 2.350 × 10^11^ virions/mL), and 5.00% (*w*/*w*) (formulations 4–5, corresponding to approximately 7.833 × 10^11^ virions/mL). Periodically, these dispersions were stirred and allowed to rest for at least 1 h, so complete hydration of the sodium alginate could occur. To start alginate polymerization via release of the calcium ions dispersed in the formulations, hydrolysis solutions (fresh aqueous GDL) were added and quickly homogenized with the polymeric dispersions previously prepared, and the resulting mixtures immediately transferred into the molds. Alginate polymerization via internal gelification was performed for 72 h at room temperature, with the resulting hydrogel matrices being maintained at 4 °C until use. Table 1 displays the final compositions of the calcium alginate hydrogel formulations containing entrapped phage particles.

#### 2.2.4. Evaluation of the Maintenance of Lytic Viability of the Immobilized Phage Particles

The influence of the biopolymeric matrix components upon formation of bacterial lawns, structural cohesion of the polymerized matrices, and maintenance of lytic viability of the immobilized phages was assessed for all formulations. All microbiological assays consisted of preparing a *P. aeruginosa* lawn and putting the bio-hydrogels in contact with the lawn with subsequent incubation at 37 °C for 24 to 48 h, after a macroscopical analysis was performed to both the matrices and the bacterial lawns.

#### 2.2.5. Tomographic Analyses via X-ray Transmission

Tomographic models of the biopolymeric hydrogels were obtained in a computed X-ray transmission tomograph [52] from Bruker microCT (model SkyScan 1174, Kontich, Belgium). The hydrogel samples were attached to a hollow support inside the tomograph chamber, and image slices were gathered at 34 kV and 529 μA. For obtaining the tomographic images, a large number of image slices of the object positions. The samples were rotated with angular increments of 0.8°, producing 225 radiographs (projections) per image, with each integrating 1304 × 1304 (width × height) pixels with a spatial resolution of 9.761 μm. An exposure of 3000 ms per projection was performed. At the exit of the X-ray source, one used an Al filter with 0.25 mm thickness. For reconstructing the three-dimensional tomographic images of the hydrogel samples, via an appropriate composition of bi-dimensional images, mathematical algorithms were used. The three-dimensional images had 1304 × 1304 × 1304 pixels and the same spatial resolution of their bi-dimensional counterparts, thus, making the volume of data generated for each sample isotropic with relation to the spatial resolution. With all the projections at hand (viz. radiographs obtained at each angular position), one used the software NRecon™ from Bruker (vs. 1.6.9.4, Kontich, Belgium) with the algorithm of Reference [53] for reconstructing the tomographic images, whereas the software CTVox™ (vs. 2.6.0 r908-64bit, from Bruker microCT), CTan™ (vs. 1.13.5.1-64bit, from Bruker microCT), and CTvol (vs. 2.2.3.0-64bit, from Bruker microCT) were used for processing the tomographic images.

#### 2.2.6. Scanning Electron Microscopy Analyses

The surface characteristics and the internal morphology of the biopolymeric hydrogels were analysed in a dispersive-energy scanning electron microscope (DESEM) from LEO Electron Microscopy/Oxford (model Leo 440i, Cambridge, UK) coupled with an EDS (Energy Dispersive X-ray) detector (model 6070, Cambridge, UK). Samples of the biopolymeric hydrogel were either cut or cryo-fractured and sputter-coated with an Au film (200 Å thickness) via cathodic pulverization in a metalizing device (Sputter Coater) from EMITECH (model K450, Kent, UK). Photomicrographs were produced using electron beams with accelerations of 20 keV and electric current of 100 mA.

#### 2.2.7. Phage-Based Bacterial Biosensing Trials Using Bio-Detection System I

**Figure 1:**
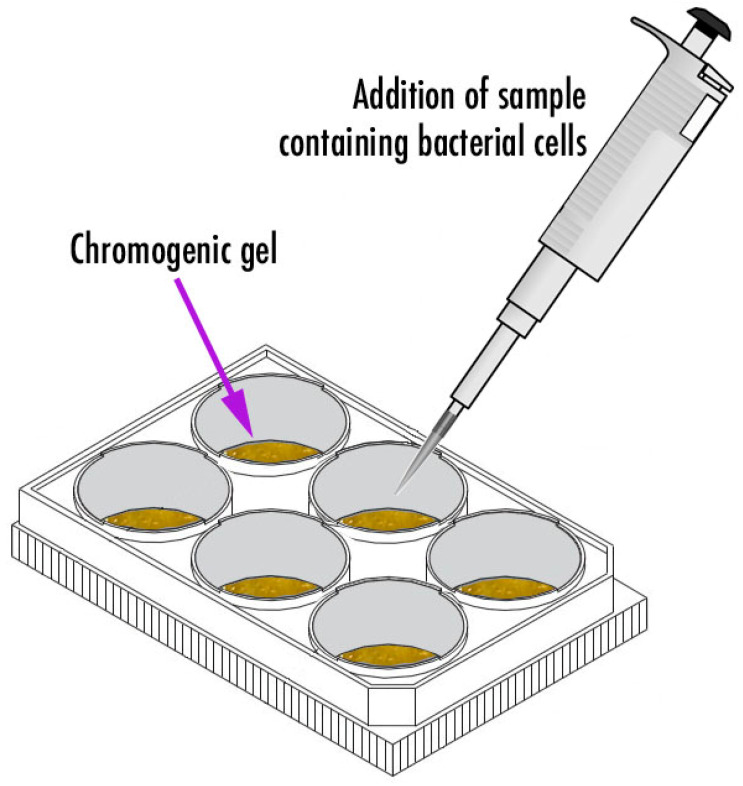
Bio-detection system I, consisting of a six-well culture plate within which the chromogenic formulations integrating a cocktail of phage particles and sodium 1,2-naftoquinone-4-sulfonate together with specific amounts of gelatin and casein were poured and allowed to polymerize.

The system depicted in Figure 1 was designed and utilized. It consisted of a six-well culture plate within which the chromogenic formulations were poured and allowed to polymerize (formulations 1–3, Table 1).

Three chromogenic gel formulations were prepared according to the data in Table 1 (viz. formulations 1–3). On top of each bio-hydrogel corresponding to formulations 1–3 (Table 1), 2 mL of *P. aeruginosa* bacterial suspension were added, corresponding to the addition of ca. 5.66 × 10^7^ CFU/mL (or 1.132 × 10^8^ cells) to the surface of each bio-hydrogel. The chromogels were formulated so as to produce a putative bioreaction following phage-mediated lysis of the bacterial cells, viz.: (casein + gelatin) + cytoplasmic enzymes released upon phage-mediated cell lysis → glutamic acid + proline $\overset{\mathrm{sodium-}1,2\mathrm{-naphthoquinone-}4\mathrm{-sulfonate},\;\;\;\mathrm{pH}\approx 8.0}{\to }$ orange chromophores (presence of proline) or green chromophores (presence of glutamic acid). The chromogenic gel that produced the best results in terms of color change was selected for further studies. Of the selected formulation, six identical chromogels were prepared in a six-well culture plate and, on top of them, different volumes of *P. aeruginosa* bacterial suspension (containing a given amount of bacterial cells, viz. 1.560 × 10^8^ cells, 7.800 × 10^7^ cells, 3.900 × 10^7^ cells, 1.950 × 10^7^ cells, 9.750 × 10^6^ cells, and 4.875 × 10^6^ cells) were applied. As a control for these bacterial biosensing experiments, the chromogel formulation selected on the basis of its better responsiveness was also tested by applying 1.000 × 10^7^ cells of *S. aureus* CCCD-S009 (2 mL of a bacterial suspension containing 5.0 × 10^6^ CFU/mL) on its surface. The bacterial biosensing assays were carried out during a timeframe of 6 h.

#### 2.2.8. Phage-Based Bacterial Biosensing Trials Using a Bio-Detection System II

**Figure 2:**
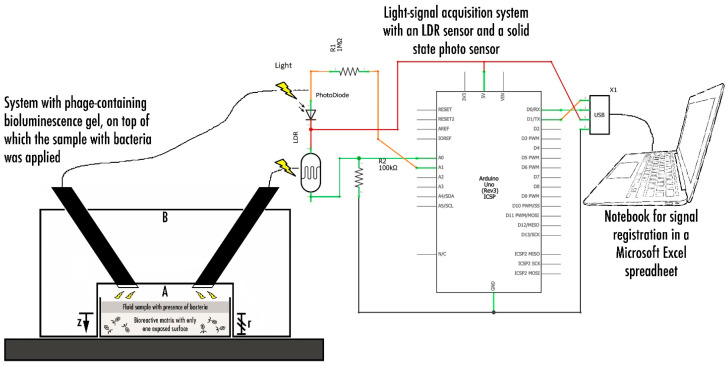
Bio-detection system II, consisting of a small cylindrical equipment machined in rigid white PVC, having approximately 6 cm in height and 6 cm in diameter, divided into a lower part (**A**) where the bio-reactive hydrogel integrating the immobilized phage cocktail, luciferin, luciferase, ADP (adenosine 5′-diphosphate sodium salt), and Mg^2+^ is housed, and an upper part (**B**) housing the light sensors, which was used to cover (**A**). The heart of the system designed consisted of a receptacle for the biopolymeric matrix containing the phage cocktail, above which the light sensors were attached and connected to an Arduino platform, which, in turn, was connected via a USB port to a notebook computer.

The system depicted in Figure 2 was designed and utilized. In order to produce a relationship between the light/luminescence intensity developed in the bioluminescent hydrogel and the bacterial load (*P. aeruginosa*) present in a biological fluid in contact with the surface of the bio-hydrogel, optical sensors were integrated with a signal transducer in the bio-detection kit. It consisted of small cylindrical equipment machined in rigid, white PVC, which was approximately 6 cm in height and 6 cm in diameter. It was divided into a lower part (A), where the hydrogel (Table 1, formulations 4–5) integrating the immobilized phage cocktail is housed, and an upper part (B), housing the light sensors, covered (A). Hence, the heart of the system designed consisted of a receptacle (A) for the biopolymeric matrix containing the phage cocktail, above which the light sensors (LDR with 4 mm in diameter from Advanced Photonix Inc. (Ann Arbor, MI, USA). InGaAs semiconductor photodiode from Phograin Technology Co., Ltd. (Shenzhen, China)) were attached and connected to an Arduino platform, which, in turn, was connected via a USB (Universal Serial Bus) port to a notebook computer.

On top of the bio-hydrogel corresponding to formulations 4–5 (Table 1), 1 mL of *P. aeruginosa* suspension was added, corresponding to the addition of ca. 4.66 × 10^7^ cells to the surface of each bio-hydrogel. The bioluminescent hydrogels were formulated so as to produce a putative bioluminescent bioreaction following phage-mediated lysis of the bacterial cells, viz.: (phage cocktail)_in the gel_ + luciferin_in the gel_ + firefly luciferase_in the gel_ + ADP_in the gel_ + Mg^2+^_in the gel_ + methylparaben_in the gel_ + (cytoplasmic adenylate kinase + ATP)_released upon cell lysis_ → AMP + PPi (inorganic pyrophosphate) + oxyluciferin + CO_2_ + hν (bioluminescence). To increase the amount of bioluminescence, producing a more intense (positive) signal, the cytoplasmic adenylate kinase released upon phage-mediated cell lysis would react with free (added) ADP to increase the amount of ATP produced, hence, amplifying the amount of ATP. Hence, more ATP in the presence of firefly luciferase would (at least in theory) produce a stronger bioluminescent signal. An electronic system was designed and implemented, integrating two light sensors with different sensibilities, viz. a 4-mm LDR (light-dependent resistor) sensor and a solid state photosensor, so as to detect any bioluminescence upon addition of *P. aeruginosa* suspension to the bio-reactive hydrogel.

## 3. Results

In the research work described herein, we propose two bio-detection systems for *P. aeruginosa*, based on a cocktail of phage particles structurally and functionally stabilized within a biopolymeric matrix endowed with chromogenic/bioluminescent characteristics. Three lytic phages were isolated from a hospital effluent in Sorocaba (Brazil), and elected on the basis of their ability to form clear and dimensionally different plaques of lysis, which is an imprint feature of virulent phage particles.

Burst of the host bacterial cell induced “from within” by the virion progeny is the culmination of the lytic cycle of replication and, in addition to the release of virion progeny, lysis of the infected host cell also releases intracellular components that can be advantageously exploited as markers for the purpose of bacterial bio-detection [1,54,55]. This was the basis for the research work undertaken.

The three different phages used in this work, viz. phages ph0031, ph0034, and ph0041, were fully characterized in a previous work by Harada et al. (currently submitted for publication). Plating serial dilutions of concentrated phage suspensions allowed the determination of average phage particle concentrations of 4.40 × 10^13^ virions/mL, 1.20 × 10^12^ virions/mL, and 1.80 × 10^12^ virions/mL, for phages ph0031, ph0034, and ph0041, respectively.

### 3.1. Lytic Activity of the Isolated Phages

**Figure 3:**
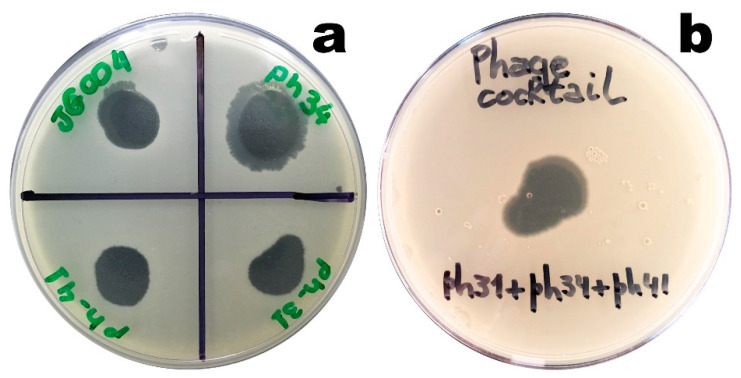
Results obtained following evaluation of the lytic activity of the isolated phage particles ph0031, ph0034, and ph0041, and a comparison with the lytic activity of phage JG004 obtained from the DSMZ collection (**a**) and of the lytic activity of the phage cocktail produced with the three isolated phage particles (**b**) on a *P. aeruginosa* DSM19880 bacterial lawn.

The lytic activity of the isolated phages was evaluated to assess their infectious potential, and the results obtained are displayed in Figure 3.

The lytic activity of the isolated phages was assessed by visual inspection of lysis clearing zones, i.e., the ability of the phages to infect the bacterial host cell(s). As can be observed by inspection of Figure 3, the presence of clear lysis zones following the addition of 10 µL droplets of undiluted phage suspension (Figure 3a) or 10 µL of the phage cocktail (produced by mixing 10 µL of each concentrated phage suspension) produced thereof (Figure 3b) and incubated overnight indicated the lytic activity of each phage on the host bacterium. All lysis zones were categorized as clear [56,57].

Previous results by Harada et al. (currently submitted for publication) had indicated that the isolated phages were tailed, belonging to the order *Caudovirales* and most likely to the families *Myoviridae* (phages ph0031 and ph0034) and *Siphoviridae* (phage ph0041).

### 3.2. Evaluation of the Structural and Functional Stabilization of the Immobilized Phage Particles

**Figure 4:**
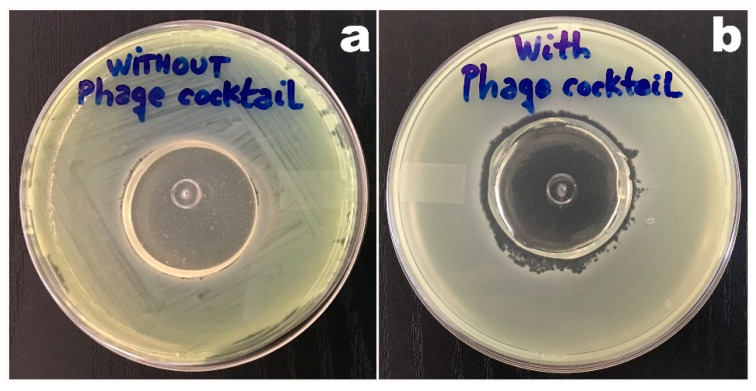
Results from evaluation of the lytic activity of the immobilized phages following integration within the bio-reactive polymeric matrix with concomitant structural and functional stabilization. (**a**) Bio-reactive polymeric matrix devoid of phage particles. (**b**) Bio-reactive polymeric matrix integrating the cocktail of phage particles.

Stabilization of the phage particles within the biopolymeric matrices, from the points of view of both their structure and function, was achieved via entrapment of the phage particles integrating the lytic cocktail in the biopolymeric matrices prior to gelification/polymerization, with a uniform spatial distribution in the matrix. All phage viability assays consisted of placing the biopolymeric matrix, one without a phage cocktail and another integrating the phage cocktail, on top of a *P. aeruginosa* DSM19880 bacterial lawn in a Petri plate, followed by incubation of the plates at 37 °C for 24–48 h. After this time period, a macroscopical analysis was carried out on the plates, to observe the presence of bacterial lysis zones promoted by the immobilized phage particles. The macroscopic aspect of the bacterial lawn was observed for the two biopolymeric matrices, having the biopolymeric matrix devoid of phage particles as a control. The results obtained are displayed in Figure 4.

From inspection of the two calcium alginate biopolymeric matrices depicted in Figure 4, it was not possible to observe any lysis zone in the bacterial lawn promoted by the control bio-polymerix matrix (Figure 4a). On the contrary, lysis of the bacterial lawn (transduced by the presence of clear zones) below and surrounding the biopolymeric matrix integrating the phage cocktail was most evident (Figure 4b), leading to the conclusion that the gelification/polymerization process did not interfere with the lytic activity of the entrapped phage particles.

### 3.3. Structural Microanalysis of the Bioluminescent/Chromogenic Hydrogel

**Figure 5:**
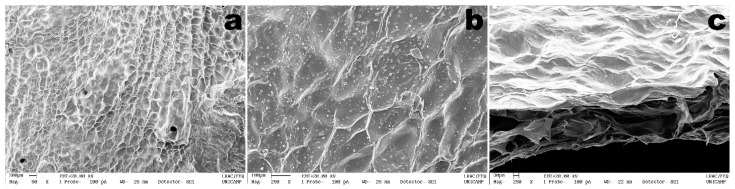
DESEM (dispersive-energy scanning electron microscopy) photomicrographs of the bioluminescent/chromogenic hydrogel surface ((**a**) ×50, (**b**) ×250, and of the cross-section fracture zone (**c**) ×250).

The DESEM images of both the surface and fractured cross-section of the bioluminescent/chromogenic hydrogel allows observation of a rugged surface without cracks (Figure 5). Since the hydrogel had to be lyophilized prior to sputter coating with colloidal gold, its surface appeared to possess cell-like structures, once filled with water.

Nevertheless, from the results obtained in the DESEM analyses performed to the bioluminescent/chromogenic hydrogel, a uniform morphology was observed. From observation of the fractured cross-section of the biopolymeric matrix (Figure 5c), a highly porous matrix structure can be clearly seen between two thicker layers. A highly uniform topography in the bioluminescent biostructure displayed in Figure 5 can also be observed.

### 3.4. Tomographic Analyses

**Figure 6:**
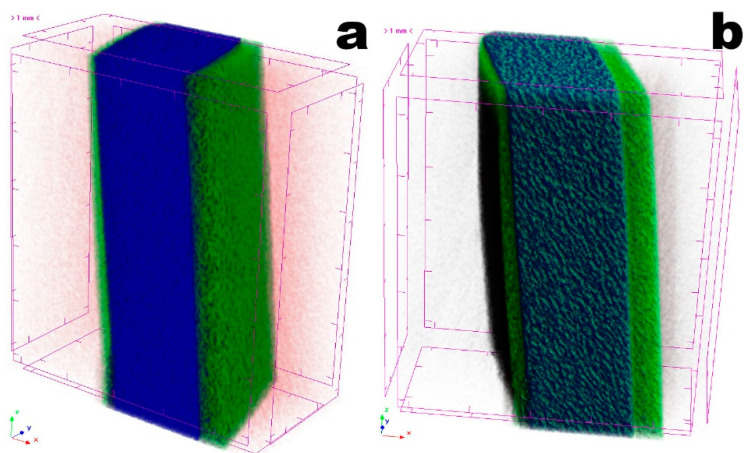
Images obtained by X-ray tomographic analyses of the bioluminescent/chromogenic hydrogel, being (**a**) a leaning surface view allowing us to observe the top side (right thicker surface in green), and (**b**) a leaning surface view allowing us to observe the bottom side (left thin surface in green).

The optimized bioluminescent/chromogenic matrix may be viewed as a natural polymer composite exhibiting a very special porous microstructure that endows the hydrogel with outstanding permeability properties. From the tomographic analyses performed to a square section of the biopolymeric matrix (Figure 6), a homogeneous and porous surface can also be observed. Three zones are evident in the bioluminescent/chromogenic hydrogel. The left (thinner) and right (thicker) layers are rich in water (in green), while the center layer (bluish) contains less water and more biopolymers. This was a very interesting observation, since the phage particles were entrapped throughout the biopolymeric matrix. Hence, the presence of a moist surface in the bioluminescent/chromogenic hydrogel promotes a better penetration of bacterial (*P. aeruginosa*) cells once a contaminated liquid sample is applied on its surface.

Due to its higher atomic density, the bio-polysaccharide matrix (in blue and green in Figure 6) absorbs more radiation, whereas void spaces appear in black. A comparative analysis of the porosity of the bioluminescent/chromogenic hydrogel is displayed in Table 2, resulting from bi-dimensional and three-dimensional morphological analyses.

### 3.5. Phage-Based Bacterial Biosensing Trials Using Biodetection System I

**Figure 7:**
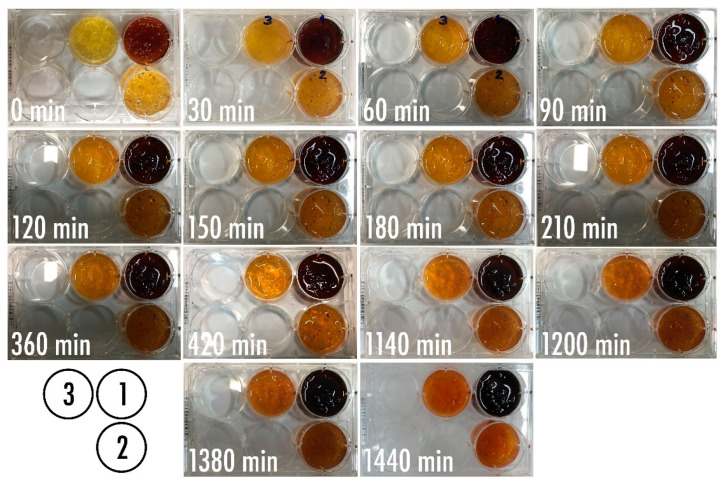
Results obtained in the optimization of the amounts of phage particles, sodium 1,2-naftoquinone-4-sulfonate, gelatin, and casein, leading to the bio-reactive chromogel for bio-detection system I, displaying the time evolution of the color produced using a fixed (added) amount of *P. aeruginosa* cells. The composition of formulations 1, 2, and 3 is displayed in Table 1.

Optimization of the bio-detection system designed to produce a putative colorimetric bioreaction following phage-mediated lysis of the bacterial cells present in a fluid directly applied over it, led to the results displayed in Figure 7.

**Figure 8:**
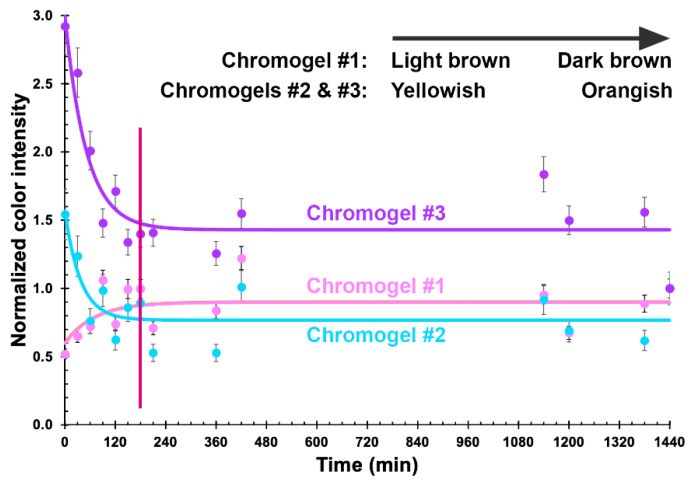
Normalized integrated color density of the three chromogels containing phage particles, sodium 1,2-naftoquinone-4-sulfonate, gelatin, and casein, further contacted with a fixed amount of *P. aeruginosa* cells, throughout the bioreaction time, allowing us to select an optimized bio-reactive chromogel. Values represent the mean of three independent assays. Error bars represent the standard deviation.

Using the freely available, open source, image processing software ImageJ (Fiji, vs. 2.0.0-rc-68/1.52e) from the National Institute of Health (USA), the surface area of each chromogel in Figure 7 was analyzed pertaining to its integrated color density, and the results duly normalized by the integrated density of the chromogels for the last reaction time assayed (Figure 8).

**Figure 9:**
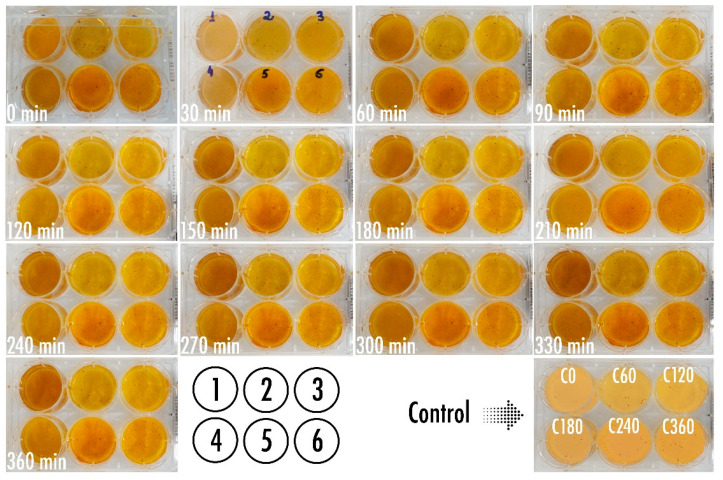
Results obtained using the selected bio-reactive chromogel with optimized concentrations of phage particles, sodium 1,2-naftoquinone-4-sulfonate, gelatin, and casein, in bio-detection system II, with evolution throughout the time of the color produced using variable added amounts of *P. aeruginosa* cells. As a control for the colorimetric bioreaction, cells of *S. aureus* CCCD-S009 were also contacted with the selected bio-reactive chromogel (Figure 9, bottom right).

The results displayed in Figure 8 allow us to clearly observe that the chromogel corresponding to formulation 3 exhibited the highest decrease in normalized integrated density during the first 180 min of contacting the bacterial cells present in the fluid directly applied over it, meaning a more pronounced color change due to phage-mediated cell lysis. Chromogel 2 exhibited the same trend, but not so pronounced. Regarding chromogel 1, a reverse trend was observed, most likely due to the initial darker color of the biopolymeric matrix, which negatively impacted visualization of the color changes. Hence, the chromogel resulting from formulation 3 (Table 1 and Figure 8) was selected for further studies. Of the selected formulation, viz. formulation 3, six identical chromogels were prepared (Figure 9) in a six-well culture plate and a different amount of bacterial cells was applied on top of each chromogel, viz. 1.560 × 10^8^ cells (chromogel 1, top lef), 7.800 × 10^7^ cells (chromogel 2, top middle), 3.900 × 10^7^ cells (chromogel 3, top right), 1.950 × 10^7^ cells (chromogel 4, bottom left), 9.750 × 10^6^ cells (chromogel 5, bottom middle), and 4.875 × 10^6^ cells (chromogel 6, bottom right). The development of color was followed every 30 min up to 360 min of the experiment.

As a control for these bacterial biosensing experiments, the chromogel selected on the basis of its better responsiveness, viz. the one evolving from formulation 3, was also applied with 1.000 × 10^7^ cells of *S. aureus* CCCD-S009 (2 mL of a bacterial suspension containing 5 × 10^6^ CFU/mL, bottom right in Figure 9) on its surface, and any color change was followed after 0, 60, 120, 180, 240, and 360 min of contact of the hydrogel with the cells of *S. aureus* CCCD-S009. No growth of this bacteria could be observed during the timeframe of the assay, viz. 6 h. The objective of the chromogel system developed was to attain bacterial detection within a short timeframe, and, therefore, longer times of the assay were not interesting for the intended purposes.

**Figure 10:**
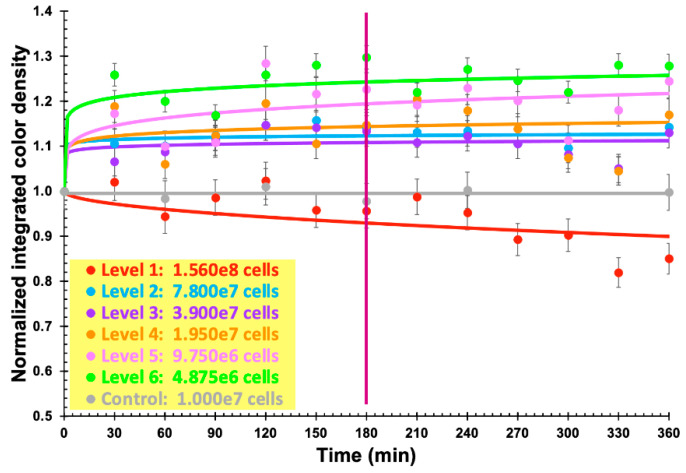
Normalized integrated color density of the optimized bio-reactive chromogel integrating phage particles, sodium 1,2-naftoquinone-4-sulfonate, gelatin, and casein, further added with variable amounts of *P. aeruginosa* cells, throughout the bioreaction time. Cells of *S. aureus* CCCD-S009 were used as a control for the bio-detection assays. Values represent the mean of three independent assays. Error bars represent the standard deviation.

Using the same image processing software ImageJ (Fiji, vs. 2.0.0-rc-68/1.52e), the surface area of each chromogel was again analyzed pertaining to its integrated color density, and the results were duly normalized by the integrated color density of the chromogels immediately after addition of the bacterial suspension (Figure 10).

### 3.6. Phage-Based Bacterial Biosensing Trials Using Biodetection System II

**Figure 11:**
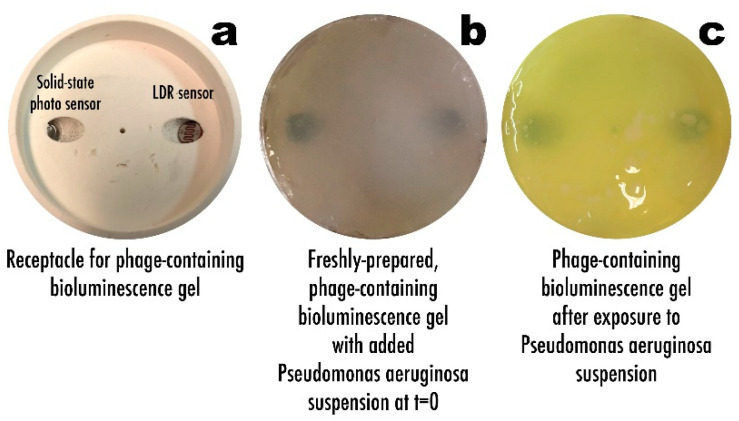
Receptacle for the bio-reactive hydrogel in bio-detection system II, showing the position of the two light sensors (**a**), macroscopic aspect of the freshly-prepared phage-containing bio-reactive hydrogel (**b**), and macroscopic aspect of the phage-containing bio-reactive hydrogel with produced bioluminescence following exposure to *P. aeruginosa* cells (**c**).

Figure 11 displays the biosensor receptacle for the bioluminescent bio-hydrogel (Figure 11a) allowing to observe the position of the two light sensors employed, a solid-state photosensor, and an LDR (Light-Dependent Resistor) sensor. The macroscopic aspect of the freshly-prepared bioluminescent bio-hydrogel with added *P. aeruginosa* suspension at t = 0 can be observed in Figure 11b, whereas the same bioluminescent bio-hydrogel with added *P. aeruginosa* suspension after a 3-h timeframe assay can be observed in Figure 11c.

**Figure 12:**
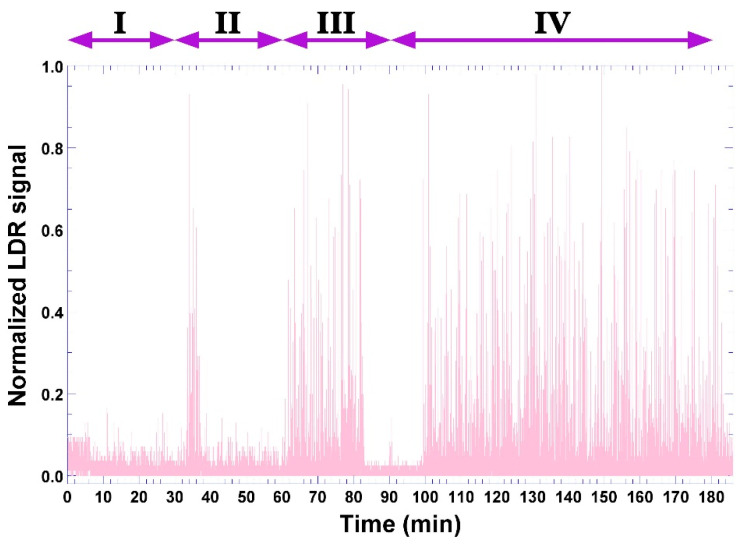
Evolution of the normalized LDR (Light-Dependent Resistor) signal throughout the bioreaction timeframe, using the bioluminescent hydrogel in bio-detection system II.

During this timeframe, the bio-hydrogel produced according to formulation 5 (Table 1) developed bioluminescence (Figure 11c), whose signal was captured by the LDR sensor (Figure 2 and Figure 11a) and, with the aid of a computer program written in C++ language and transmitted to the Arduino platform (Figure 2), produced the results depicted in Figure 12 in the form of normalized LDR signals (of original arbitrary units).

Four major moments can be observed in the signal acquired by the LDR sensor (Figure 12). Moment I may be attributed to diffusion of bacterial cells into just below the surface of the bio-hydrogel, not so deep, thereby contacting with some phage particles and, after 30 min, some of the bacterial cells being lysed with concomitant release of cytoplasmic contents and bio-reacting with the constituents of the bio-hydrogel, leading to the first intense bioluminescent signals between 34–38 min, which is a result that is in close agreement with the general duration of a lytic cycle. This includes replication of the phage genome within the bacterial cytoplasm with concomitant synthesis of viral proteins and assembly of more lytic phage virions in cycles of ca. 30 min each, which, with the help of phage-synthesized holins and lysins, causes lysis of the bacterium and releases a wave of newly formed virions. Moment II followed after another 30 min, now producing a more intense and durable bioluminescent signal (moment III) between 62–82 min. The onset of moment III is in close agreement with previous findings by Balcão et al. [8] in the sense that it would take about 60 min for *P. aeruginosa* cells to penetrate 3 mm deep into the calcium alginate hydrogel core. Between 100–182 min, moment IV clearly illustrates the phage amplification by a massive infection of the bacterial cells with concomitant lysis and bioreaction of cytoplasmic contents with the reactants added to the bio-hydrogel formulation, producing durable luminescence in the bio-hydrogel until the end of the assay timeframe.

**Figure 13:**
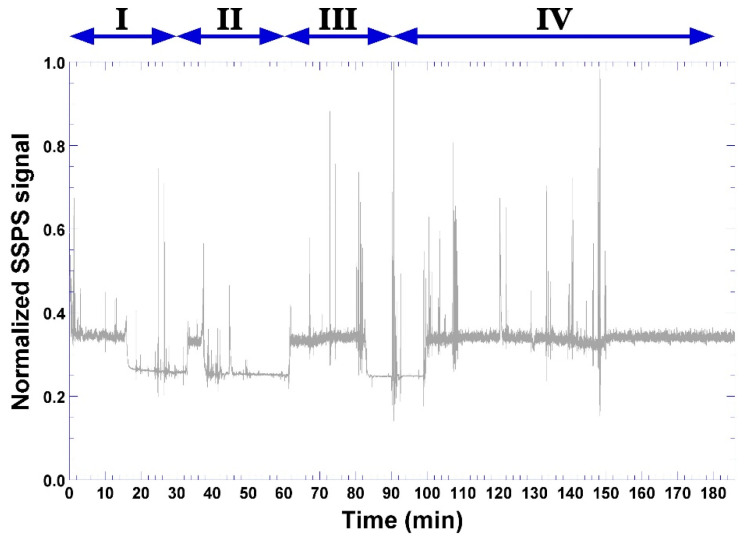
Evolution of the normalized solid-state photosensor signal throughout the bioreaction timeframe using the bioluminescent hydrogel in bio-detection system II.

The results obtained simultaneously from the solid-state photo sensor (photodiode) are displayed in Figure 13.

As can be seen from the inspection of Figure 13, the signal acquired by the photodiode also allows us to observe the same four phases, but it was somewhat erratic without a clear correspondence with the signal from the LDR sensor.

## 4. Discussion

Based on their performance, all isolated phages produced clear lysis zones in the *P. aeruginosa* DSM19880 bacterial lawn, with phages ph0031 (lower right quadrant in Figure 3a), ph0034 (upper right quadrant in Figure 3a), and ph0041 (lower left quadrant in Figure 3a), producing lysis zones with diameters that were ca. 93%, 107%, and 93% of that produced by phage JG004 (upper left quadrant in Figure 3a), respectively. In addition, a secondary halo can be observed in the lysis zone produced by phages ph0034 and JG004, indicative of the production of phage depolymerases. These results were indicative that the cocktail joining the three isolated phages was suitable for integrating the bacterial bio-sensing polymeric matrices. High titre suspensions (10^13^ (phage ph0031) and 10^12^ (phages ph0034 and ph0041) PFU/mL) were obtained for the three phages.

According to previous results by Harada et al. (currently submitted for publication), the isolated phages belong to the order Caudovirales, since they are tailed, with phages ph0031 and ph0034 belonging most likely to the *Myoviridae* family and phage ph0041 belonging most likely to the *Siphoviridae* family.

Analyzing the two calcium alginate biopolymeric matrices depicted in Figure 4, lysis of the bacterial lawn (transduced by the presence of clear zones) surrounding the biopolymeric matrix integrating the phage cocktail was highly evident (Figure 4b), allowing us to conclude that the immobilized phage particles retained their lytic activity following the gelification/polymerization process. Despite the fact that the microbiological assays have revealed maintenance of the lytic viability of the immobilized phages, the theoretical micro-architecture of the alginate matrix with a low pore interconnectivity may, in fact, impact negatively the access of the phage particles to their migrating bacterial host, by retaining the phage particles within the polymerized alginate matrix and promoting a lower diffusivity of the bacterial cells into the inner core of the bio-hydrogel. Hence, the bacterial lysis observed in the *P. aeruginosa* DSM19880 lawn may have been a result of the lytic activity of the phages dispersed in the more superficial layers of the calcium alginate matrices. Nevertheless, the phage particles retained their lytic activity upon immobilization within the polymerized matrix, which is a result only possible following their full structural and functional stabilization.

Physical immobilization (entropic confinement) of phage particles within an entangled polymeric matrix rich in water molecules, such as the one prevailing in hydrogels, promotes alterations in the thermodynamic conditions of the nano-environment surrounding each phage particle, drastically decreasing water molecule motions in their micro-neighborhood and leading to enhanced thermodynamic stability [2,58,59,60]. The net result is an increase in the translational, rotational, and vibrational viscosities of the phage particles, with concomitant rigidification of their three-dimensional architecture and consequent decrease of entropy, hence, promoting structural and functional stabilization [1,2,58,59]. Such stabilization of the phage particles was, in fact, observed in a previous research work [7], with the hydrogel maintaining its lytic activity during several months (data not shown).

The DESEM analyses performed for the bioluminescent/chromogenic hydrogels revealed a highly porous matrix between two thicker layers (Figure 5), which facilitate mobility of the bacterial cells upon contact of the bioluminescent/chromogenic hydrogel with aqueous samples containing *P. aeruginosa* cells.

The results from X-ray tomographic analyses made to the hydrogels (Figure 6) closely agree with those obtained by DESEM (Figure 5), which indicated that the bioluminescent/chromogenic hydrogel possessed a highly homogeneous and porous biopolymeric matrix. These are clearly important and positive results since such high porosity did not entangle the entrapped phage particles, making them readily available for meeting the bacterial cells contacting the surface of the biopolymeric matrix.

Anisotropy is related to a material being directionally dependent and being defined as a difference in the properties of a material when measured along different directions, as opposed to isotropy, which implies identical material properties in all directions. The degree of anisotropy, calculated as $1-\left\{\left({\mathrm{Eigenvalue}}_{\min}\right)/\left({\mathrm{Eigenvalue}}_{\max}\right)\right\}$, is 0 for total isotropy and 1 for total anisotropy. As can be observed from inspection of the data in Table 2, the degree of anisotropy is 0.6936 for the bioluminescent/chromogenic hydrogel, a value that is clearly closer to anisotropy than to isotropy. Since the bioluminescent/chromogenic hydrogel can be considered an engineered biomaterial with an interconnected biopolymeric network, this result is easily understandable by observing the DESEM images displayed in Figure 5.

An analysis of the structure of the bioluminescent/chromogenic hydrogel allows one to find that it has almost equal values of open and total porosities (Table 2). Adding to this, the mean fragmentation index (Table 2) is related to connectivity and produces a value of relative convexity or concavity of the total object surface with concavity indicating connectivity (and the presence of “nodes”) and convexity indicating isolated disconnected structures (“struts”). Lower fragmentation indexes are related to better connected lattices while higher fragmentation indexes are related to a more disconnected structure. While scarce enclosed cavities and concave surfaces can push the fragmentation index to (large) positive values, the results obtained for our bioluminescent/chromogenic hydrogel were large and negative (Table 2), which are consistent with the DESEM analyses performed. The importance of these results is due to the fact that phage particles were dispersed homogeneously within the biopolymeric matrix of the bioluminescent/chromogenic hydrogel during polymerization, and, therefore, the hydrogel can be contacted with a biological fluid containing bacterial cells with either of its surfaces.

As can be seen from inspection of Figure 7, inclusion of a higher amount of sodium 1,2-naftoquinone-4-sulfonate (SNQS) together with higher amounts of gelatin and casein led to production of a very dark-brown chromogel, whose color changes were not so perceptible during the timeframe of the assays. On the other hand, integration of the lowest amounts of SNQS, gelatin and casein, in the biopolymeric matrix led to production of a light-yellowish chromogel, whose color changes over time were more pronounced following phage-mediated lysis of the bacterial cells present on a fluid directly applied over it (Figure 7).

As apparent from inspection of the results displayed in Figure 7, the colorimetric bioreaction produced within the chromogels led to the release of proline, which, in turn, resulted in orange chromophores conferring the orangish color to the bio-hydrogels. The chromogel resulting from formulation 3 (Table 1 and Figure 8) was selected for further studies. The bioreaction resulting from the hydrolysis of casein/gelatin by the cytoplasmic enzymes released upon phage-mediated cell lysis led to the production of the imino-acid proline (possessing a secondary amine, called an imine, instead of a primary one), which reacted quantitatively and stoichiometrically with SNQS and produced orange chromophores. This is a reaction that is in close agreement with reports by Troll [61]. As apparent from the inspection of the results displayed in Figure 9, the colorimetric bioreaction produced within the chromogels led to release of proline, which, in turn, resulted in visible color changes arising from the orange chromophores produced, conferring an orangish color to all bio-hydrogels with varying degrees of intensity along the assay timeframe and depending on the initial number of bacterial cells applied on top of them.

The results displayed in Figure 10 allow us to clearly observe that increasing the amount of bacterial cells contacting the chromogel leads to a negative trend of the normalized integrated color density in the chromogel. This observation might closely agree with the mucoid appearance of *P. aeruginosa* bacterial lawn on calcium alginate hydrogels, at high bacterial concentrations, likely due to either production of alginate by the bacterial cells themselves or to the activity of lyases produced by the bacterial cells causing degradation of the alginate chains with concomitant reduction of viscosity [7,51], thereby, leading to the decrease in normalized integrated color density observed (Figure 10). On the contrary, for a lower number of bacterial cells in contact with the biopolymeric chromogel, the normalized integrated color density increases by 20% after initiating contact of the bacterial cells with the surface of the chromogel, and remains virtually constant throughout the remaining timeframe of the assay. For bacterial cell numbers up to 10^7^, no visible change could be observed beyond 180 min of assay (Figure 10), allowing us to infer that the presence of this bacterium in a fluid could be detected within a 3 h timeframe assay. When using cells of *S. aureus* CCCD-S009 as a control for these bacterial biosensing experiments, the chromogel selected on the basis of its better responsiveness, viz. the one evolving from formulation 3, maintained its color throughout the assay timeframe (Figure 10), indicating that no bioreaction could be observed (Figure 10) since the lytic phages entrapped within the chromogel were strictly lytic and specific for *P. aeruginosa* cells. These results were, once again, consistent with previous results of the host range determination for the isolated phages by Harada et al. (currently submitted for publication). Additionally, no growth of this bacteria could be observed during the timeframe of the assay, viz. 6 h.

When testing the biodetection system II, the difference in the aspect of the bioluminescent biohydrogel before and after the assay timeframe was most clear with the bioluminescent bio-hydrogel contacting with *P. aeruginosa* suspension becoming somewhat fluorescent/bioluminescent after 180 min of contact with the bacterial cells. In the bioluminescent bio-hydrogel with added *P. aeruginosa* suspension depicted in Figure 11c, a slightly mucoid appearance on its surface could be observed, most likely due to exopolysaccharide (likely alginate) production by the *P. aeruginosa* bacterial cells [7,8,51] (Figure 11).

According to the mathematical simulations performed by Balcão et al. [8] for this very kind of bio-hydrogel matrix, porosity highly influences penetration of *P. aeruginosa* cells into the core of the hydrogels. It was also observed by the same researchers that more porous hydrogels become saturated after 3 h of exposure to the bacteria.

For the diffusion of bacterial cells in a porous matrix, such as the chromogenic/bioluminescent hydrogels developed, the diffusion process occurs in the frontier between two extremes, viz. bulk diffusion vs. Knudsen diffusion [8], since the magnitude of the mean free path of the bacteria has the same order of magnitude of the mean pore diameter in the matrix network. Similarly to the work developed by Balcão et al. [8], this was the case with the biopolymeric hydrogels developed in the present research work. On average, self-propelled *P. aeruginosa* cells have a much greater motion speed than that provoked by spontaneous fluctuation within the medium [62].

In their work, Balcão and colleagues [8] obtained the value D_bact,eff_ = 1.290 × 10^−10^ m^2^/s for the effective diffusion coefficient for *P. aeruginosa* bacterial cells in a similar calcium alginate hydrogel, which is a value that is of the same order of magnitude as that obtained by Liu et al. [63] for *Cellulosimicrobium cellulans* GS6, concluding that it takes about 1 h for *P. aeruginosa* cells to penetrate 3 mm deep into the calcium alginate hydrogel core. Balcão and colleagues [8] inferred that a bio-detection device using such an alginate hydrogel would respond quite rapidly.

As time elapses, bacterial cells that swarmed deeper into the bio-hydrogel core become entrapped and thermodynamically more stable, which can be attributed to a thermodynamic change in the microenvironment surrounding each bacterial cell due to the high reduction in the movements of aqueous solvent molecules in their micro-neighborhood within the core of the biopolymeric matrix [2,8,58], resulting in increased rotational, translational, and vibrational viscosities of the bacterial cells and, consequently, in a more rigid three-dimensional architecture with a concomitant decrease of entropy [58].

According to Balcão et al. [8], the calcium alginate hydrogel integrates pores with both larger diameters and larger throat diameters, which may facilitate a more rapid and deeper penetration of the bacterial cells into its core, since the bacterial cells may be considered equivalent to macromolecules in terms of both diffusion behavior and molecular diffusion [63].

During alginate polymerization, hyper-entanglements form within the inner core of the hydrogel matrix, which, associated with increased local viscosity, rapidly reduce bacteria mobility and penetration and prevents a homogeneous dispersion within the hydrogel [8,64].

Increasing cell density leads to an increased bacterial diffusion coefficient, which can be attributed to the hydrodynamic disturbances caused by neighboring swimming bacteria [65]. During swarming, *P. aeruginosa* cells produce a bio-surfactant at high cell densities as a *quorum sensing* response and extract wetting liquid from the surface of the polymeric matrix, with both the movement of individual cells and the swarm expansion being aided by changes in the physical properties prevailing within and on the surface of the thin liquid film recently developed [8].

The bio-detection system designed to produce bioluminescence following phage-mediated lysis of the bacterial cells present on a fluid directly applied over it, produced the results depicted in Figure 11c with formulation 5 (Table 1), integrating higher amounts of luciferase, ADP, and Mg^2+^ in the biopolymeric matrix, since the bio-hydrogel designed, according to formulation 4, did not produce any results within 180 min of the assay.

As can be observed by inspecting Figure 12, four major moments can be defined in the signal acquisition by the LDR sensor. Moment I (Figure 12) may be attributed to diffusion of bacterial cells into just below the surface of the bio-hydrogel, not so deep, thereby contacting some phage particles and, after 30 min, some of the bacterial cells being lysed with concomitant release of intracytoplasmic contents and bio-reacting with the constituents of the bio-hydrogel, leading to the first intense bioluminescent signals between 34–38 min (Figure 12). This is a result in close agreement with the general duration of a lytic cycle [1]. This includes replication of the phage genome within the bacterial cytoplasm with concomitant synthesis of viral proteins and assembly of more lytic phage virions in cycles of ca. 30 min each which, with the help of phage-synthesized holins and lysins, causes lysis of the bacterium [1,66,67] and releases a wave of newly formed virions. Moment II followed after another 30 min, now producing a more intense and durable bioluminescent signal (moment III) between 62–82 min. The onset of moment III is in close agreement with previous findings by Balcão et al. [8], in the sense that it would take about 60 min for *P. aeruginosa* cells to penetrate 3 mm deep into the calcium alginate hydrogel core. Between 100–182 min, moment IV clearly illustrates the phage amplification by a massive infection of the bacterial cells with concomitant lysis and bioreaction of intracytoplasmic contents with the reactants added to the bio-hydrogel formulation, producing durable luminescence in the bio-hydrogel until the end of the assay timeframe.

As can be seen by inspecting Figure 13, the signal emanating from the photodiode was somewhat erratic, without a clear correspondence with the signal from the LDR sensor. Hence, the photodiode sensor was deemed not suitable for the intended purpose of the bacterial bio-detection system II.

The bacterial bio-detection system II was designed to capture extremely low luminous intensities. For this, two sensors possessing distinct characteristics were employed, viz. a 4-mm LDR sensor and a 3 × 4 mm photodiode. The LDR sensor is typically composed of a CdS filament, whose resistivity varies with the intensity of incident light [68]. The spectral sensitivity range is approximately 400–900 nm, which is more efficient in the range equivalent to the human eye, making the LDR sensor recommended to capture yellow-greenish luminescence [68]. The other sensor used was a solid-state photodiode, where the incident light is directed to the P-N junction of the semiconductor, generating an electric current to be picked up by the circuit. The sensitivity of the solid-state photodiode is broad, and maximum in the infrared region, but with a relative response above 0.5 for the visible spectrum [68].

The Arduino electronic prototyping platform [69], composed of a microprocessor, digital and analog inputs and outputs, and a USB serial communication port, was used for the sensor signal processing system. The electronic scheme used for bio-detection system II is illustrated in Figure 2. The resistors selected and used were such that they maximized both the sensitivity and the detection range for each sensor. The LDR and photodiode signals (two points per second from each sensor) were sent to the input ports A0 and A1 (Figure 2), processed by a program written in C++, and sent numerically to the USB serial port, from where they followed the computer via a serial terminal. The data captured in the terminal was then copied periodically to a data-processing program (viz. Microsoft Excel). The results displayed in Figure 8, Figure 10 and Figure 12 allow us to conclude that both biosensing devices were able to positively detect the presence of *P. aeruginosa* cells within a 3 h timeframe assay.

In a previous research work by Harada et al. (currently submitted for publication), the host range and efficiency of plating (EOP) of the isolated phages revealed that phages ph0031, ph0034, and ph0041 were able to bind and infect several strains of *P. aeruginosa* isolated from clinical isolates (data not shown), killing the bacteria (between 33% (phage ph0034) and 89% (phages ph0031 and ph0041) of those strains). Although the host range was wide, the EOP of the phages for those strains was generally low, likely because the phages were not able to produce a large amount of progeny virions. Nevertheless, the bio-detection systems designed and tested in this research work should be able to detect those (and other) strains from clinical isolates.

The screening of common pools often associated with *P. aeruginosa* and its rapid and specific diagnosis have important epidemiological significance for the prevention and control of infections related to this bacterial. Hence, the uniquely high specificity of phages for a given bacterial species, along with a short life cycle, makes these metabolically inert entities useful not only for treating bacterial infections but also for biological detection of that pathogen.

## 5. Conclusions

In this research work, we propose two bio-detection systems for *P. aeruginosa*, based on a cocktail of three different lytic phages isolated from environmental sources in Brazil, which were structurally and functionally stabilized within a biopolymeric matrix. Based on their lytic performance, all phages produced clear zones of lysis in *P. aeruginosa* lawns, indicative that the cocktail joining the three isolated phages was suitable for integrating the bacterial biosensing polymeric matrices. All phage particles isolated were tailed and, hence, dsDNA phages, with two of them belonging to the *Myoviridae* family and one belonging to the *Siphoviridae* family. The immobilized phage particles were viable and retained their lytic activity after the polymerization process, leading to bio-reactive hydrogels, thus allowing us to conclude that stabilization of both their structure and function was fully attained. Using bio-detection system I, for bacterial cell numbers up to 10^7^ CFU, no visible change could be observed beyond 180 min of the assay, allowing us to infer that the presence of this bacterium in a fluid could be detected within a 3 h timeframe assay using this biosensing device. Using bio-detection system II, bioluminescence was developed when contacting the hydrogel with the bacterium, whose signal was successfully captured by an LDR sensor and transformed into numerical values with the aid of a computer program written in C++ language and transmitted to an Arduino platform. From the bioluminescence signals captured by the LDR sensor, four major moments could be defined that were in close agreement with the general duration of a lytic cycle, and allowed to positively detect a (*P. aeruginosa*) bacterial load in a timeframe of less than 180 min. Regarding the signal emanating from the photodiode sensor, it was somewhat erratic without a clear correspondence with a signal from the LDR sensor, and, hence, the photodiode sensor was deemed not suitable for the intended purpose of bacterial bio-detection system II. The results obtained in the present research effort allow us to conclude that both (viz. chromogenic and bioluminescent) biosensing devices were able to positively detect the presence of *P. aeruginosa* cells within a 3 h timeframe assay. The use of bio-reactive hydrogels facilitates handling, and, hence, are desirable for integrating a bioanalytical system with practical use. In the research work described herein, the bio-reactive matrices were intended for stabilizing phage particles and exhibit chromogenic/bioluminescent properties, following penetration of *P. aeruginosa* cells supposedly present in (biological) fluids contacting a single surface of the hydrogel, and a reaction with the stabilized phage particles.

## Figures and Tables

**Table 1:** Final compositions of the bacterial bio-detection systems encompassing calcium alginate biopolymeric matrices containing entrapped phage particles.

Component	Bio-Detection System I			Bio-Detection System II	
	Formulation 1	Formulation 2	Formulation 3	Formulation 4	Formulation 5
Phage cocktail [% (*w*/*w*), m (mg)]	0.0045; 0.045 (45 µL)	0.0045; 0.045 (45 µL)	0.0045; 0.045 (45 µL)	0.050; 0.150 (150 µL)	0.050; 0.150 (150 µL)
Methylparaben [% (*w*/*w*), m (mg)]	0.000966; 0.0966	0.000968; 0.0968	0.000969; 0.0969	0.0010; 0.0300	0.0010; 0.0300
Sodium alginate [% (*w*/*w*), m (mg)]	1.50; 150	1.50; 150	1.50; 150	1.50; 45.00	1.50; 45.00
CaCO_3_ 22.5 mM [% (*w*/*w*), m (mg)]	0.2250; 22.50	0.2250; 22.50	0.2250; 22.50	0.2233; 6.700	0.2233; 6.700
GDL 48 mM [% (*w*/*w*), m (mg)]	0.8600; 86.00	0.8600; 86.00	0.8600; 86.00	0.8600; 25.80	0.8600; 25.80
Casein [% (*w*/*w*), m (mg)]	0.1000; 10.0	0.0500; 5.00	0.0100; 1.00	-	-
Gelatin [% (*w*/*w*), m (mg)]	0.1000; 10.0	0.0500; 5.00	0.0100; 1.00	-	-
Sodium 1,2-naftoquinone-4-sulfonate [% (*w*/*w*), m (mg)]	0.1300; 13.0	0.0130; 1.30	0.0100; 1.00	-	-
Luciferin [% (*w*/*w*), m (mg)]	-	-	-	0.1667; 5.00	0.1667; 5.00
Luciferase [% (*w*/*w*), m (mg)]	-	-	-	0.1667; 5.00	0.3333; 10.0
ADP [% (*w*/*w*), m (mg)]	-	-	-	0.00006667; 0.002	0.0003333; 0.010
Mg^2+^ [% (*w*/*w*), m (mg)]	-	-	-	0.00006667; 0.002	0.0006667; 0.020
Ultrapure water [% (*w*/*w*), m (mg)]	97.08; 9708	97.30; 9730	97.38; 9738	97.10; 2913	96.93; 2908
TOTAL [% (*w*/*w*), m (mg)]	100; 10000	100; 10000	100; 10000	100; 3000	100; 3000

**Table 2:** Results obtained from the X-ray tomographic analyses performed to the bioluminescent/chromogenic hydrogel.

Parameter	Bioluminescent/Chromogenic Hydrogel Morphological Analysis	
	Bi-Dimensional (2D)	Three-Dimensional (3D)
Number of layers	-	101.000
Pixel size (µm)	9.7610	9.7610
Total VOI (volume of interest), TV (mm^3^)	6.8340	6.8292
Object volume, Obj.V (mm^3^)	3.55432	3.4115
Percent object volume, Obj.V/TV (%)	52.0094	49.9542
Total VOI surface, TS (mm^2^)	28.2910	27.6702
Object surface, Obj.S (mm^2^)	432.0516	377.5217
Intersection surface, i.S (mm^2^)	-	7.5075
Object surface/volume ratio, Obj.S/Obj.V (mm^−1^)	121.5566	110.6616
Cross-sectional thickness, Cs.Th (mm)	0.02533	-
Object surface density, Obj.S/TV (mm^−1^)	-	55.2802
Degree of anisotropy, DA	-	3.2631 (0.6936)
Eigenvalue 1	-	3.5861
Eigenvalue 2	-	6.5491
Eigenvalue 3	-	11.7020
Number of closed pores, Po.N(cl)	-	5125
Volume of closed pores, Po.V(cl) (mm^3^)	-	0.00472
Surface of closed pores, Po.S(cl) (mm^2^)	-	2.7836
Closed porosity (percent), Po(cl) (%)	6.8883	0.1383
Mean fragmentation index, Fr.I (mm^−1^)	−58.7603	−71.3382
Mean fractal dimension, FD	1.7048	2.6834
Volume of open pore space, Po.V(op) (mm^3^)	-	3.4130
Open porosity (percent), Po(op) (%)	-	49.9766
Total volume of pore space, Po.V(tot) (mm^3^)	-	3.4178
Total porosity (percent), Po(tot) (%)	-	50.0458
Euler number, Eu.N	-	−72,773.000
Connectivity, Conn	-	83,998.000
Connectivity density, Conn.Dn (mm^−3^)	-	12,299.7496

## Data Availability

The data presented in this study is contained within the article.

## References

[1] Harada L.K., Silva E.C., Campos W.F., Del Fiol F.S., Vila M.M.D.C., Dąbrowska K., Krylov V.N., Balcão V.M. (2018). Biotechnological applications of bacteriophages: State of the art. Microbiol. Res..

[2] Rios A.C., Vila M.M.D.C., Lima R., Del Fiol F.S., Tubino M., Teixeira J.A., Balcão V.M. (2018). Structural and functional stabilization of bacteriophage particles within the aqueous core of a W/O/W multiple emulsion: A potential biotherapeutic system for the inhalational treatment of bacterial pneumonia. Process Biochem..

[3] Caselli D., Cesaro S., Ziino O., Zanazzo G., Manicone R., Livadiotti S., Cellini M., Frenos S., Milano G.M., Cappelli B. (2010). Multidrug resistant Pseudomonas aeruginosa infection in children undergoing chemotherapy and hematopoietic stem cell transplantation. Haematologica.

[4] Neves M.T., Lorenzo M.E.P., Almeida R.A.M.B., Fortaleza C.M.C.B. (2010). Antimicrobial use and incidence of multidrug-resistant Pseudomonas aeruginosa in a teaching hospital: An ecological approach. Rev. Soc. Bras. Med. Trop..

[5] Chan B.K., Abedon S.T., Loc-Carrillo C. (2013). Phage cocktails and the future of phage therapy. Future Microbiol..

[6] Haq I.U., Chaudhry W.N., Akhtar M.N., Andleeb S., Qadri I. (2012). Bacteriophages and their implications on future biotechnology: A review. Virol. J..

[7] Balcão V.M., Moreira A.R., Moutinho C.G., Chaud M.V., Tubino M., Vila M.M.D.C. (2013). Structural and functional stabilization of phage particles in carbohydrate matrices for bacterial biosensing. Enzym. Microb. Technol..

[8] Balcão V.M., Barreira S.V.P., Nunes T.M., Chaud M.V., Tubino M., Vila M.M.D.C. (2014). Carbohydrate hydrogels with stabilized phage particles for bacterial biosensing: Bacterium diffusion studies. Appl. Biochem. Biotechnol..

[9] Balcão V.M., Glasser C.A., Chaud M.V., del Fiol F.S., Tubino M., Vila M.M.D.C. (2014). Biomimetic aqueous-core lipid nanoballoons integrating a multiple emulsion formulation: A suitable housing system for viable lytic bacteriophages. Colloids Surf. B Biointerfaces.

[10] Puapermpoonsiri U., Spencer J., van der Walle C.F. (2009). A freeze-dried formulation of bacteriophage encapsulated in biodegradable microspheres. Eur. J. Pharm. Biopharm..

[11] Sulakvelidze A., Kutter E., Kutter E., Sulakvelidze A. (2005). Bacteriophage therapy in humans. Bacteriophages: Biology and Applications.

[12] Sulakvelidze A., Alavidze Z., Morris J.G. (2001). Bacteriophage therapy. Antimicrob. Agents Chemother..

[13] Hanlon G.W. (2007). Bacteriophages: An appraisal of their role in the treatment of bacterial infections. Int. J. Antimicrob. Agents.

[14] Abedon S.T. (2006). The Bacteriophages.

[15] Chibani-Chennoufi S., Bruttin A., Dillmann M.L., Brüssow H. (2004). Phage-host interaction: An ecological perspective. J. Bacteriol..

[16] Rohwer F. (2003). Global phage diversity. Cell.

[17] Hatfull G.F., Hendrix R.W. (2011). Bacteriophages and their genomes. Curr. Opin. Virol..

[18] Pope W.H., Hatfull G.F. (2015). Adding pieces to the puzzle: New insights into bacteriophage diversity from integrated research-education programs. Bacteriophage.

[19] Hwang W., Yoon S.S. (2019). Virulence Characteristics and an Action Mode of Antibiotic Resistance in Multidrug-Resistant Pseudomonas aeruginosa. Sci. Rep..

[20] Ribeiro Á.C.S., Crozatti M.T.L., Silva A.A., Macedo R.S., Machado A.M.O., Silva A.T.A. (2020). Pseudomonas aeruginosa in the ICU: Prevalence, resistance profile, and antimicrobial consumption. J. Braz. Soc. Trop. Med..

[21] Abadi A.T.B., Rizvanov A.A., Haertlé T., Blatt N.L. (2019). World Health Organization Report: Current Crisis of Antibiotic Resistance. Bionanoscience.

[22] Schmelcher M., Loessner M.J., Zourob M., Elwary S., Turner A.P.F. (2008). Bacteriophage: Powerful tools for the detection of bacterial pathogens. Principles of Bacterial Detection: Biosensors, Recognition Receptors and Microsystems.

[23] Schmelcher M., Loessner M.J. (2014). Application of bacteriophages for detection of foodborne pathogens. Bacteriophage.

[24] Smartt A.E., Ripp S. (2011). Bacteriophage reporter technology for sensing and detecting microbial targets. Anal. Bioanal. Chem..

[25] Smietana M., Bock W.J., Mikulic P., Ng A., Chinnappan R., Zourob M. (2011). Detection of bacteria using bacteriophages as recognition elements immobilized on long-period fiber gratings. Opt. Express.

[26] Weinstein M.P., Mirrett S., Reller L.B. (1988). Comparative evaluation of Oxoid Signal and BACTEC radiometric blood culture systems for the detection of bacteremia and fungemia. J. Clin. Microbiol..

[27] Pheiffer C., Carroll N.M., Beyers N., Donald P., Duncan K., Uys P., van Helden P. (2008). Time to detection of Mycobacterium tuberculosis in BACTEC systems as a viable alternative to colony counting. Int. J. Tuberc. Lung Dis..

[28] Rees C.E.D., Loessner M.J., Sulakvelidze E.K.A. (2005). Phage for the detection of pathogenic bacteria. Bacteriophages: Biology and Applications.

[29] Rees C.E., Dodd C.E. (2006). Phage for rapid detection and control of bacterial pathogens in food. Adv. Appl. Microbiol..

[30] Mole R.J., Maskell T.W.O. (2001). Phage as a diagnostic—The use of phage in TB diagnosis. J. Chem. Technol. Biotechnol..

[31] Pai M., Kalantri S.P. (2005). Bacteriophage-based tests for tuberculosis. Indian J. Med. Microbiol..

[32] Hagens S., Loessner M.J. (2007). Application of bacteriophages for detection and control of foodborne pathogens. Appl. Microbiol. Biotechnol..

[33] Ripp S. (2010). Bacteriophage-based pathogen detection. Adv. Biochem. Eng. Biotechnol..

[34] Heo J., Hua S.Z. (2009). An overview of recent strategies in pathogen sensing. Sensors.

[35] Capek P., Kirkconnell K.S., Dickerson T.J. (2010). A Bacteriophage-based platform for rapid trace detection of proteases. J. Am. Chem. Soc..

[36] Carlson K., Kutter E., Sulakvelidze A. (2004). Working with bacteriophages: Common techniques and methodological approaches. Bacteriophages Biology and Applications.

[37] Pinheiro L.A.M., Pereira C., Frazão C., Balcão V.M., Almeida A. (2019). Efficiency of Phage φ6 for Biocontrol of Pseudomonas syringae pv. syringae: An in Vitro Preliminary Study. Microorganisms.

[38] Pinheiro L.A.M., Pereira C., Barreal M.E., Gallego P.P., Balcão V.M., Almeida A. (2019). Use of phage ϕ6 to inactivate Pseudomonas syringae pv. actinidiae in kiwifruit plants: In vitro and ex vivo experiments. Appl. Microbiol. Biotechnol..

[39] Shinomiya T., Shiga S. (1979). Bactericidal activity of the tail of Pseudomonas aeruginosa bacteriophagePS17. J. Virol..

[40] Markoishvili K., Tsitlanadze G., Katsarava R., Glenn J., Sulakvelidze A. (2002). A novel sustained-release matrix based on biodegradable poly(ester amide)s and impregnated with bacteriophages and an antibiotic shows promise in management of infected venous stasis ulcers and other poorly healing wounds. Int. J. Dermatol..

[41] Petty N.K., Evans T.J., Fineran P.C., Salmond G.P.C. (2006). Biotechnological exploitation of bacteriophage research. Trends Biotechnol..

[42] McNerney R., Mallard K., Urassa H.M.R., Lemma E., Donoghue H.D. (2007). Colorimetric phage-based assay for detection of rifampin-resistant Mycobacterium tuberculosis. J. Clin. Microb..

[43] Azzazy H.M.E., Highsmith W.E. (2002). Phage display technology: Clinical applications and recent innovations. Clin. Biochem..

[44] Mosier-Boss P.A., Lieberman S.H., Andrews J.M., Rohwer F.L., Wegley L.E., Breitbart M. (2003). Use of fluorescently labeled phage in the detection and identification of bacterial species. Appl. Spectrosc..

[45] Ashida A., Yamada Y., Kamidate T. (2008). Colorimetric method for enzymatic screening assay of ATP using Fe(III)-xylenol orange complex formation. Anal. Bioanal. Chem..

[46] Park J.K., Chang H.N. (2000). Microencapsulation of microbial cells. Biotechnol. Adv..

[47] Covizzi L.G., Giese E.C., Gomes E., Dekker R., Silva R. (2007). Imobilização de células microbianas e suas aplicações biotecnológicas. Ciências Exatas Tecnológicas.

[48] Moutinho C.G., Matos C.M., Teixeira J.A., Balcão V.M. (2012). Nanocarrier possibilities for functional targeting of bioactive peptides and proteins: State-of-the-art. J. Drug Target..

[49] Souza G.R., Christianson D.R., Staquicini F.I., Ozawa M.G., Snyder E.Y., Sidman R.L., Miller J.H., Arap W., Pasqualini R. (2006). Networks of gold nanoparticles and bacteriophage as biological sensors and cell-targeting agents. Proc. Natl. Acad. Sci. USA.

[50] Draget K.I., Moe S.T., Skjak-Bræk G., Smidsrod O., Stephen A.M., Phillips G.O., Williams P.A. (2006). Alginates. Food Polysaccharides and Their Applications.

[51] Donati I., Paoletti S., Rehm B.H.A. (2009). Material Properties of Alginates. Alginates: Biology and Applications.

[52] Oliveira J.M., Martins A.C.G. (2009). Construction and test of low cost X-ray tomography scanner for physical-chemical analysis and nondestructive inspections. AIP Conf. Proc..

[53] Feldkamp L.A., Davis L.C., Kress J.W. (1984). Practical cone-beam algorithm. J. Opt. Soc. Am. A.

[54] Ahmed A., Rushworth J.V., Hirst N.A., Millner P.A. (2014). Biosensors for whole-cell bacterial detection. Clin. Microbiol. Rev..

[55] Zourob M., Ripp S., Zourob M. (2010). Bacteriophage-Based Biosensors. Recognition Receptors in Biosensors.

[56] Mirzaei M.K., Nilsson A.S. (2015). Isolation of phages for phage therapy: A comparison of spot tests and efficiency of plating analyses for determination of host range and efficacy. PLoS ONE.

[57] Melo L.D.R., Sillankorva S., Ackermann H.-W., Kropinski A.M., Azeredo J., Cerca N. (2014). Isolation and characterization of a new Staphylococcus epidermidis broad-spectrum bacteriophage. J. Gen. Virol..

[58] Balcão V.M., Vila M.M.D.C. (2015). Structural and functional stabilization of protein entities: State-of-the-art. Adv. Drug Deliv. Rev..

[59] Campos W.F., Silva E.C., Oliveira T.J., Oliveira J.M., Tubino M., Pereira C., Vila M.M.D.C., Balcão V.M. (2020). Transdermal permeation of bacteriophage particles by choline oleate: Potential for treatment of soft-tissue infections. Future Microbiol..

[60] Silva E.C., Oliveira T.J., Moreli F.C., Harada L.K., Vila M.M.D.C., Balcão V.M. (2020). Newly isolated lytic bacteriophages for Staphylococcus intermedius, structurally and functionally stabilized in a hydroxyethylcellulose gel containing choline geranate: Potential for transdermal permeation in veterinary phage therapy. Res. Vet. Sci..

[61] Troll W. (1953). The reaction of naphthoquinone-4-sulfonate with imino acids. J. Biol. Chem..

[62] Thonemann P.C., Evans C.J. (1976). The dispersal of an initial concentration of motile bacteria. J. Gen. Microbiol..

[63] Liu C.-H., Wu J.-Y., Chang J.-S. (2008). Diffusion characteristics and controlled release of bacterial fertilizers from modified calcium alginate capsules. Bioresour. Technol..

[64] Burke M.D., Park J.O., Srinivasarao M., Khan S.A. (2005). A novel enzymatic technique for limiting drug mobility in a hydrogel matrix. J. Control. Release.

[65] Wu M., Roberts J.W., Kim S., Koch D.L., DeLisa M.P. (2006). Collective bacterial dynamics revealed using a three-dimensional population-scale defocused particle tracking technique. Appl. Environ. Microbiol..

[66] Storms Z.J., Sauvageau D. (2015). Modeling tailed bacteriophage adsorption: Insight into mechanisms. Virology.

[67] Dąbrowska K., Switala-Jelen K., Opolski A., Weber-Dabrowska B., Gorski A. (2005). Bacteriophage penetration in vertebrates. J. Appl. Microbiol..

[68] Knoll F.G. (2010). Radiation Detection and Measurement.

[69] Arduino Arduino. www.arduino.cc.

